# Trueness of cone-beam computed tomography-derived skull models fabricated by different technology-based three-dimensional printers

**DOI:** 10.1186/s12903-023-03104-w

**Published:** 2023-06-16

**Authors:** Xiaotong Wang, Sohaib Shujaat, Eman Shaheen, Eleonora Ferraris, Reinhilde Jacobs

**Affiliations:** 1 OMFS-IMPATH Research Group, Department of Imaging & Pathology, Faculty of Medicine, KU Leuven & Oral and Maxillofacial Surgery, University Hospitals Leuven, Kapucijnenvoer 33, Leuven, 3000 Belgium; 2 Department of Oral and Maxillofacial Surgery, The First Affiliated Hospital of Harbin Medical University, Youzheng Street 23, Nangang, 150001 Harbin China; 3 King Abdullah International Medical Research Center, Department of Maxillofacial Surgery and Diagnostic Sciences, College of Dentistry, King Saud bin Abdulaziz University for Health Sciences, Ministry of National Guard Health Affairs, Kingdom of Saudi Arabia, Riyadh, 14611 Saudi Arabia; 4 Department of Mechanical Engineering, KU Leuven Campus De Nayer, Jan Pieter de Nayerlaan 5, 2860 Sint-Katelijne-Waver, Belgium; 5 Department of Dental Medicine, Karolinska Institutet, Alfred Nobels allé 8, 141 52, Huddinge, Sweden

**Keywords:** Printing, three-dimensional, Computer-aided design, Dimensional measurement accuracy, Tomography, Skull

## Abstract

**Background:**

Three-dimensional (3D) printing is a novel innovation in the field of craniomaxillofacial surgery, however, a lack of evidence exists related to the comparison of the trueness of skull models fabricated using different technology-based printers belonging to different cost segments.

**Methods:**

A study was performed to investigate the trueness of cone-beam computed tomography-derived skull models fabricated using different technology based on low-, medium-, and high-cost 3D printers. Following the segmentation of a patient’s skull, the model was printed by: (i) a low-cost fused filament fabrication printer; (ii) a medium-cost stereolithography printer; and (iii) a high-cost material jetting printer. The fabricated models were later scanned by industrial computed tomography and superimposed onto the original reference virtual model by applying surface-based registration. A part comparison color-coded analysis was conducted for assessing the difference between the reference and scanned models. A one-way analysis of variance (ANOVA) with Bonferroni correction was applied for statistical analysis.

**Results:**

The model printed with the low-cost fused filament fabrication printer showed the highest mean absolute error ($1.33\pm 0.24 \text{mm}$), whereas both medium-cost stereolithography-based and the high-cost material jetting models had an overall similar dimensional error of $0.07\pm 0.03 \text{mm}$ and $0.07\pm 0.01 \text{mm}$, respectively. Overall, the models printed with medium- and high-cost printers showed a significantly ($p<0.01$) lower error compared to the low-cost printer.

**Conclusions:**

Both stereolithography and material jetting based printers, belonging to the medium- and high-cost market segment, were able to replicate the skeletal anatomy with optimal trueness, which might be suitable for patient-specific treatment planning tasks in craniomaxillofacial surgery. In contrast, the low-cost fused filament fabrication printer could serve as a cost-effective alternative for anatomical education, and/or patient communication.

## Background

Recent advancements in additive manufacturing (AM), also known as three-dimensional (3D) printing and rapid prototyping (RP), have led to an ever-increasing impact on the field of craniomaxillofacial surgery [1]. The manufacturing of anatomically true skull models from cone-beam computed tomography (CBCT) and computed tomography (CT) data have been successfully employed for improving diagnostic accuracy, treatment planning and simulation of complex surgical procedures, training, and anatomical education [2]. By offering further spatial details on a patient’s anatomy and pathology, these models act as a surgical aid, increasing the accuracy of the procedure and leading to more predictable post-operative results with reduced risk of complications [3]. The main clinical applications of patient-specific 3D printed skull models include pre-bending reconstruction plates, prosthesis engineering, and fabrication of personalized surgical guides and titanium-based implants for craniomaxillofacial defects [4]. Additionally, 3D printed models also serve as a supplementary tool to improve the informed consent process and offer an effective way of communication with the patients. From an educational perspective, compared to the intangible virtual models and ethically challenged cadaveric skull models, 3D printed skull models are a key for laying a solid foundation for novices to learn the maxillofacial surgical procedures and anatomical learning [5, 6].

Currently, a wide variety of 3D printers exist in the market for printing maxillofacial skeletal models. These printers can be further classified as low-cost desktop/consumer grade and high-cost professional 3D printers [7]. Fused filament fabrication machines are among the most widely adopted low-cost/consumer grade desktop 3D printers [8]. They are mostly priced between $\$1500\!-\!\$7000$, having a build size of less than $10\times 10\times 10 \text{inches}$, layer thickness between $100\text{-}300 {\upmu }\text{m}$, $0.5\%$ dimensional tolerance normally based on calibration cube as a benchmark, slow printing process and utilize thermoplastic filaments as the main printing material, such as polylactic acid (PLA) and acrylonitrile butadiene styrene (ABS). In contrast, the majority of higher cost professional 3D printers are either selective laser sintering (SLS), selective laser melting (SLM), or UV (ultraviolet) jet-based technologies (e.g. multi jetting) for printing metals and high-performance polymers in addition to the aforementioned materials. They are priced between $\$\text{20,000-}\$\text{200,000}$ with a build size bigger than $12\times 12\times 12 \text{inches}$, layer thickness down to a few tens of microns, dimensional tolerance of 0.15% based on calibration cube, and the ability of fast and batch printing [9–11].

For preoperative planning and clinical training, craniomaxillofacial 3D models are typically manufactured via in-house or by commercial external printing service centers utilizing high-cost professional-grade 3D printers. The expertise of operators, cost and delivery time of the models might influence the patient’s treatment process, consequently, limiting their general applicability [12, 13]. In order to propel the application of 3D printers and increase their generalizability, it is essential to assess whether desktop printers can produce skull models with comparable trueness to high-cost printers. Previous studies have demonstrated that low-cost printers offer comparable trueness to that of professional ones when printing specific anatomical structures, such as the mandible and orbital region [7, 14, 15]. However, there is insufficient evidence to assess the trueness or precision of a 3D printed complete skull model consisting of craniomaxillary complex and mandible. In addition, only a few studies have utilized a 3D assessment method [7, 16]. Most studies have been dependent on landmark-based methodologies that are prone to human error and variability [17, 18]. There is a lack of evidence comparing the trueness of skull models fabricated using printers from different cost segments. Therefore, the aim of this study was to investigate the trueness of CBCT-derived skull models fabricated using different technology based on low-, medium-, and high-cost 3D printers.

## Methods

This research was conducted in compliance with the World Medical Association Declaration of Helsinki on medical research. The study was approved by the Ethical Review Board of the University Hospitals Leuven, Leuven, Belgium (reference number: S64493) for retrospectively collecting and using patient imaging data. Informed consent was obtained from all participants.

### Data acquisition

A 32-year-old female patient’s CBCT image consisting of a normal complete skull (craniomaxillary complex and mandible) without any pathological condition or artefacts was retrospectively obtained from the Dentomaxillofacial Radiology Center (University Hospitals of Leuven, Leuven, Belgium). The scanning was performed using NewTom VGI evo (Verona, Italy) at $110 \text{kV}$ tube voltage, $0.3 \text{mm}$ slice thickness and $24\times 19 \text{cm}$ field of view. The image was stored in a Digital Imaging and Communications in Medicine (DICOM) format.

### Model design

**Fig. 1:**
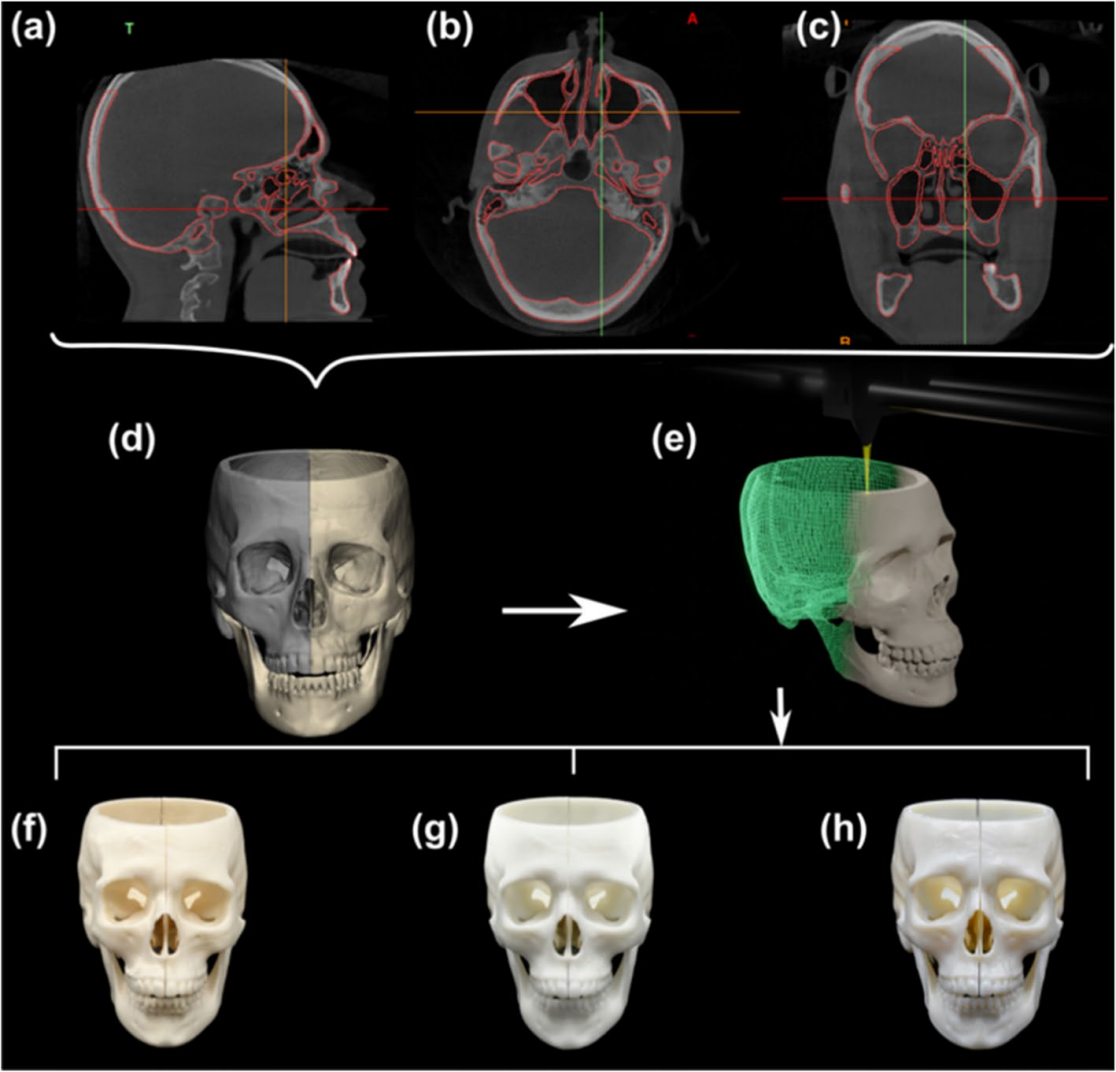
Workflow of 3D printing. **a** Segmentation of the CBCT-derived skull in sagittal view. **b** Segmentation of the CBCT-derived skull in axial view. **c** Segmentation of the CBCT-derived skull in coronal view. **d** STL of designed 3 anatomical parts: a mandible and 2 hemimaxillofacial complexes. **e** Model fabrication. **f** A photo of 3D printed low-cost FFF model by Prusa i3 MK3S. **g** A photo of 3D printed medium-cost SLA model by ShapeSolid A600. **h** A photo of 3D printed high-cost MJ model by Objet 350

The DICOM images were imported into Mimics 22.0 (Materialise, Leuven, Belgium), where thresholding-based semi-automatic segmentation of the skeletal structures was performed (Fig. 1a, b and c). Manual delineation of the bony contours was carried out to improve either the quality of overall segmentation or in situations where thresholding was not enough to sufficiently segment the regions with thin structures, such as sinuses, nasal region and margins of foramina. It involved manual addition or removal of the bone mask using eclipse and the livewire function of the software. The operator scrolled through all the slices in coronal, axial, and sagittal planes to confirm that the region of interest was completely masked without any over- or under-estimation of the margins. The segmented skull was converted to standard tessellation language (STL) format and imported into 3-Matic 14.0 (Materialise, Leuven, Belgium), where the craniomaxillary part of the skull was split at the mid-sagittal plane into two halves (Fig. 1d). The splitting was performed to allow for surface inspection of the interior parts of the skull at a later step. Additionally, snap hooks and grooves were designed to attach and detach the two segments. The final STL data for the purpose of printing consisted of left skull, right skull and mandible (Fig. 1e).

### 3D printing

Three different printing technologies and printers were utilized for the fabrication of the model: a low-cost Prusa i3 MK3S printer (Prusa research, Prague, Czech Republic; Fused Filament Fabrication, FFF technology) (Fig. 1f), a medium-cost ShapeSolid A600 printer (Lexcent, Shenzhen, China; Stereolithography, SLA technology) (Fig. 1g), and a high-cost Objet 350 printer (Stratasys, Eden Prairie, MN, USA; Material Jetting, MJ technology) (Fig. 1h). Table 1 describes the specifications of the printers and materials. The selection of material and settings were based on each company’s recommendations for printing anatomical skeletal models.

**Table 1:** Model specifications of the high, medium and low-cost printers

Printer type	Printer name, manufacturer	Printing technique	Material	Post-processing
High-cost printer	Objet Connex 350 (Stratasys, Eden Prairie, MN, USA)	MJ	VeroWhite	Pressured waterjet
Medium-cost printer	ShapeSolid A600 (Lexcent, Shenzhen,China)	SLA	DSM123 resin	Support removal, sanding, rinsing, sand-blasted
Low-cost printer	Prusa i3 MK3S (Prusa research, Prague, Czech Republic)	FFF	Prusament PLA Vanilla White	Support removal

**Fig. 2:**
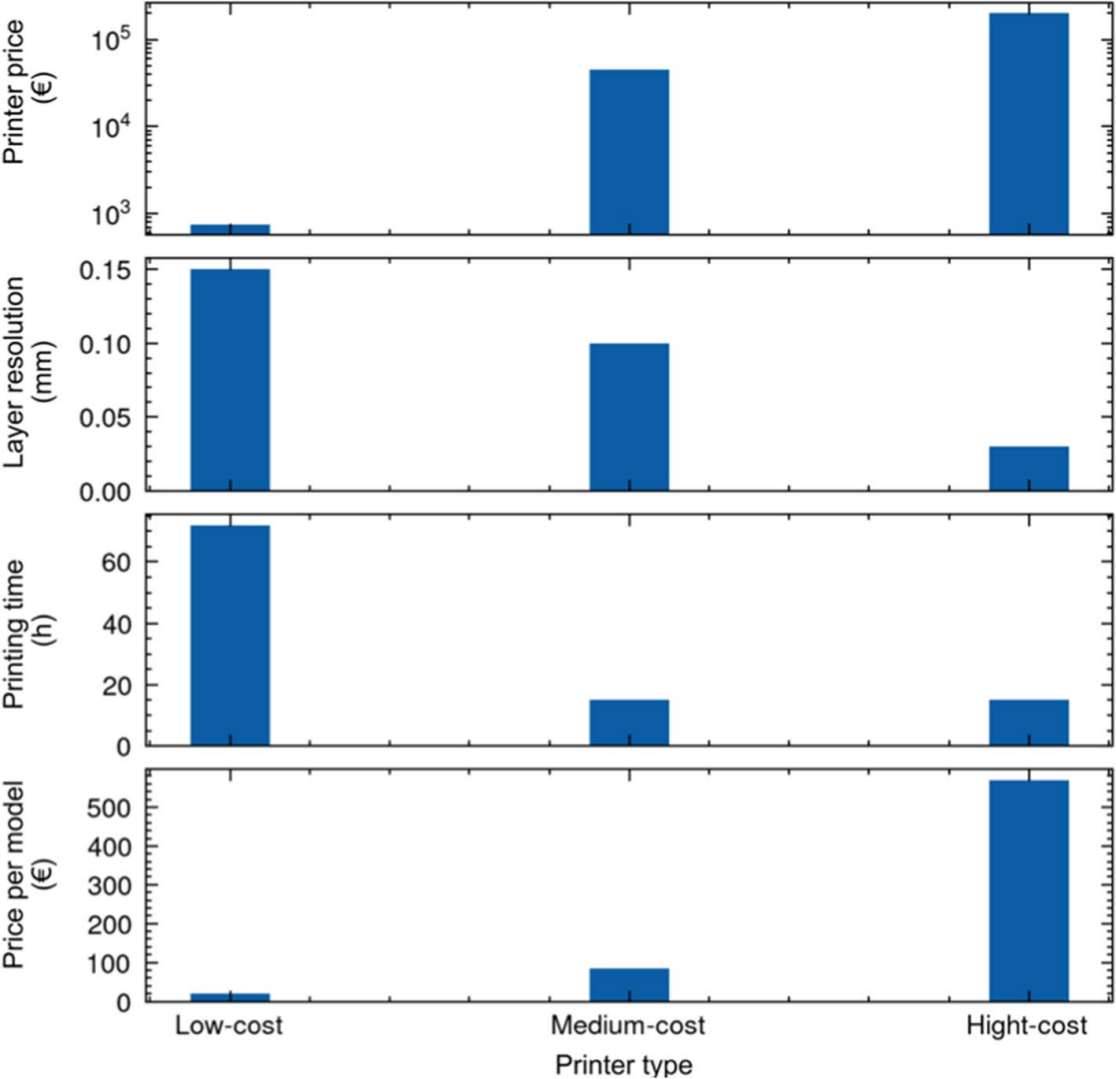
Printing parameters and cost of the low, medium and high-cost printers

Figure 2 shows the printing parameters and cost of the low, medium and high-cost printers. Each printer was used to fabricate one model (n = 3). Since the model printing had been outsourced by experienced technicians at specified printer companies, the orientation parameters and other technical and detailed cost-related parameters were not made available even upon request.

### 3D scanning and model comparison

The fabricated models were scanned with an industrial CT (Zeiss Metrotom 6 Scout system, Zeiss, US) at $140 \text{kV}$, $50 \text{W}$, $350 \text{ms}$ exposure time per picture, 3008 pictures in $360^\circ$ and $80 {\upmu }\text{m}$ original voxel size. STL files were calculated in GOM Volume Inspect (GOM Inspect, Braunschweig, Germany). Later, both the original (reference) and scanned STLs were imported into 3-Matic 14.0 (Materialise). The scanned STLs of the printed models were superimposed onto the original STL by applying surface-based registration. The registration was semi-automated in nature where the operator first added corresponding reference points onto the reference and scanned models for achieving a close alignment of the matched data in a similar 3D space. Following point-based registration, a global co-registration function with enough iterations was applied which automatically fine-tuned the registration with maximal conformance till best fit of both models was achieved without the presence of any visible spatial changes. A part comparison color-coded distance analysis was conducted for assessing the overall 3D differences or discrepancies between the surfaces of reference and scanned STLs of the printed models.

### Statistical analysis

Mean error, mean absolute error (MAE), root mean square (RMS) and volumetric values were calculated in 3-Matic 14.0 (Materialise, Leuven, Belgium), where the mean error refers to positive or negative deviation error, while MAE is the overall magnitude of error as shown in Eq. (1).

1 $$MAE\;=\;\frac1n\sum_{i=1}^n\left|x_i-x\right|$$

The volumetric error between reference and printed models was calculated using relative volumetric difference (RVD) as shown in the Eq. (2). It allows to compare the magnitude of volumetric difference regardless of the absolute values.

2 $$\mathrm{RVD}=\frac{\left|{\mathrm{Volume}}_1-{\mathrm{Volume}}_2\right|}{\left({\mathrm{Volume}}_1+{\mathrm{Volume}}_2\right)}\times100\%$$

Data were analyzed using IBM SPSS Statistics for Windows, version 21.0 (IBM Corp., Armonk, NY, USA).The Shapiro-Wilk test was used to investigate assumptions of normality. A one-way analysis of variance (ANOVA) with Bonferroni correction was applied for multiple comparisons between different printers and *p* value of $<0.05$ was considered statistically significant.

## Results

The printing process of all printers went smoothly without any issues. However, qualitative observation of the models following post-processing stage revealed that the low-cost FFF model exhibited noticeable rough patches at the right temporal region and left skull base. No other flaws were detected with the other models. Moreover, FFF model costed a fraction compared to other models, nevertheless, it required approximately 5 times longer to print.

**Fig. 3:**
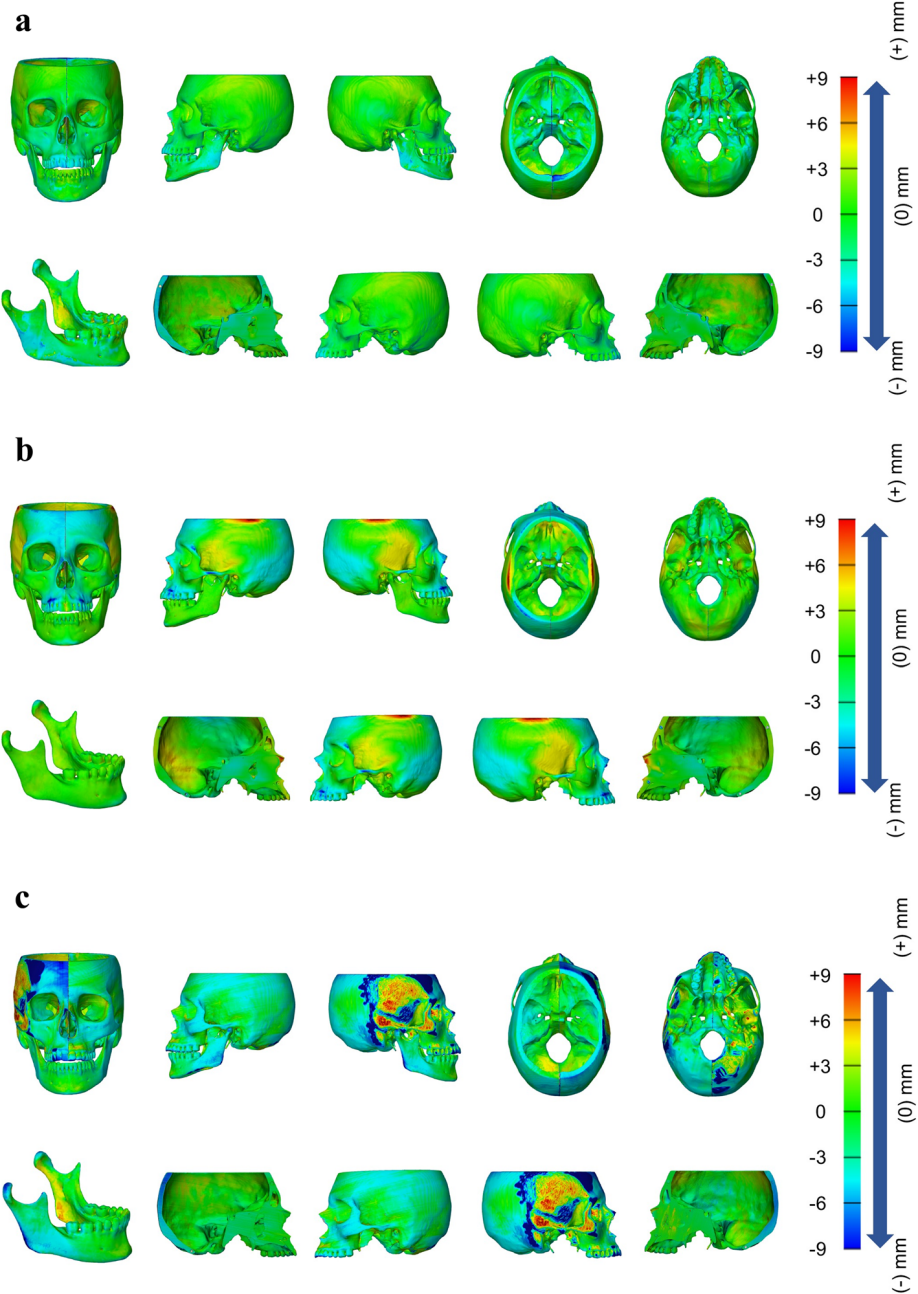
Part comparison analysis of color mapping between the reference and scanned STLs of the models printed with high-, medium-, and low-cost 3D printers. **a** High-cost MJ model by Objet 350. **b** Medium-cost SLA model by ShapeSolid A600. **c** Low-cost FFF model by Prusa i3 MK3S. From left to right: front view of skull; left view of skull; right view of skull; upper view of craniomaxillary complex; bottom view of craniomaxillary complex; side view of mandible; internal side of left skull; external side of left skull; external side of right skull; internal side of right skull

Taking into account the RMS values, the high-cost MJ, medium-cost SLA and low-cost FFF models observed an average discrepancy of $0.13\pm 0.04 \text{mm}$, $0.10\pm 0.04 \text{mm}$, and $1.69\pm 0.31 \text{mm}$, respectively (Table 2), where low-cost FFF model showed significantly higher discrepancy compared to both high-cost MJ- and medium-cost SLA-based models (p = < 0.001). Considering the overall mean absolute error, the low-cost FFF model showed the highest discrepancy ($1.33\pm 0.24 \text{mm}$), whereas both the high-cost MJ- and medium-cost SLA-based models had an overall similar dimensional error of $0.07\pm 0.01 \text{mm}$ and $0.07\pm 0.03 \text{mm}$, respectively (Fig. 3a, b and c).

**Table 2:** Summary of mean absolute error (mm) for each printed model; mean ± standard deviation values

3D printed model	Anatomy	MAE	RMS
**High-cost MJ model**	Left skull	$0.08\pm 0.14$	$0.16$
	Right skull	$0.07\pm 0.12$	$0.14$
	Mandible	$0.07\pm 0.06$	$0.09$
	Overall	$0.07\pm 0.01$	$0.13\pm 0.04$
**Medium-cost SLA model**	Left skull	$0.08\pm 0.08$	$0.11$
	Right skull	$0.09\pm 0.09$	$0.13$
	Mandible	$0.04\pm 0.04$	$0.06$
	Overall	$0.07\pm 0.03$	$0.10\pm 0.04$
**Low-cost FFF model**	Left skull	$1.17\pm 0.95$	$1.51$
	Right skull	$1.22\pm 0.91$	$1.52$
	Mandible	$1.60\pm 1.29$	$2.06$
	Overall	$1.33\pm 0.24$	$1.69\pm 0.31$

Based on the mean error, the FFF model had the highest overall surface error of $-1.23\pm 0.32 \text{mm}$ (Table 3). The medium-cost SLA model provided a more precise replica ($0.03\pm 0.01 \text{mm}$), however, slight expansion was observed at the superior margin of the skull. The high-cost MJ model demonstrated the lowest mean error ($-0.00\pm 0.03 \text{mm}$). Overall, the models printed with medium- and high-cost printers showed a significantly ($p<0.01$) lower error compared to the low-cost printer, while there were no additional significant geometrical differences found.

According to the RVD of printed anatomical structures (Table 4), the high-cost MJ and medium-cost SLA models had relatively low RVD values, ranging from 0.88 to 9.51%. In contrast, the low-cost FFF printer had significantly higher RVD values, ranging from 49.86 to 77.45% across all three anatomical structures. Overall, both MJ and SLA model showed significantly lower RVD compared to low-cost FFF models ($p<0.001$). In relation to anatomical structures, both MJ and SLA models exhibited a lower RVD for mandible compared to the craniomaxillary complex.

**Table 3:** Summary of mean error (mm) for each printer; mean ± standard deviation values

3D printed model	Anatomy	Mean	RMS
**High-cost MJ model**	Left skull	$0.01\pm 0.16$	$0.16$
	Right skull	$0.01\pm 0.14$	$0.14$
	Mandible	$-0.03\pm 0.08$	$0.09$
	Overall	$-0.00\pm 0.03$	$0.13\pm 0.04$
**Medium-cost SLA model**	Left skull	$0.02\pm 0.11$	$0.11$
	Right skull	$0.03\pm 0.12$	$0.13$
	Mandible	$0.03\pm 0.05$	$0.06$
	Overall	$0.02\pm 0.01$	$0.10\pm 0.04$
**Low-cost FFF model**	Left skull	$-1.10\pm 1.00$	$1.51$
	Right skull	$-1.00\pm 1.10$	$1.52$
	Mandible	$-1.60\pm 1.30$	$2.06$
	Overall	$-1.23\pm 0.32$	$1.69\pm 0.31$

**Table 4:** Summary of relative volumetric difference (%) for each printer

3D printed model	Anatomy	RVD
**High-cost MJ model**	Left skull	$6.19\%$
	Right skull	$9.04\%$
	Mandible	$0.93\%$
**Medium-cost SLA model**	Left skull	$6.32\%$
	Right skull	$9.51\%$
	Mandible	$0.88\%$
**Low-cost FFF model**	Left skull	$61.73\%$
	Right skull	$49.86\%$
	Mandible	$77.45\%$

## Discussion

In the era of personalized precision medicine, patient-specific 3D printed skeletal models derived from medical imaging datasets have become a standard tool [19]. An anatomically true skull is a fundamental requirement in the treatment planning workflow for complex craniomaxillofacial surgical cases and modern medical education [5]. Recent advances in the 3D printing industry have drastically lowered the price tag of the printers, however, few studies have been performed to assess whether consumer grade 3D printing technologies, such as FFF can offer a precise and true alternative to the higher-cost and professional solutions. As a result, 3D printers can be integrated into the workflows of majority of the hospitals with financial constraints. In this work, the trueness of printed skull models using a low-, a medium-, and a high-cost 3D printer was evaluated.

Due to the multi-dimensionality and complex nature of a human skull, its accurate anatomical representation is vital in all areas of craniomaxillofacial surgery [14]. The trueness of a 3D printed model is greatly dependent on the image acquisition and assessment technique [20, 21]. Since the trueness of 3D printing may be affected by the variety of imaging modalities and parameters related to slice thickness and voxel size [10], the current work employed CBCT data with a slice thickness of 0.3 mm. It would be intriguing to investigate the result of acquiring data from a pathological skull generated by a conventional CT with various scanning parameters. Traditional evaluation methods include landmark-based linear and/or angular measurements using calipers or virtual models of the printed models scanned with CT/CBCT acquisition devices [10]. In the present study, an industrial CT scanner was used to generate the surface of the printed skulls and after surface registration, part comparison analysis was employed to compare the printed skulls to the reference model. The industrial CT scanners have been known to offer higher accuracy for inspection of complex and internal features produced by additive manufacturing compared to CT/CBCT devices [22]. Furthermore, compared to traditional landmark-based methods, which are prone to human error and variability depending on the observer, the semi-automatic trueness assessment methodology applied in the present work is more reliable [23].

A previous study showed that a dimensional linear error within the range of 2% variability could be considered as clinically acceptable for the production of maxillofacial skeletal models [24]. Similarly, no studies were found assessing the clinically acceptable range of error based on 3D methodologies. It should be kept in mind that the trueness of a model is dependent on the task at hand, where higher trueness is mandatory in cases where pre-bending of reconstructive plates, surgical guide manufacturing and implant fabrication are required. Evidence suggests that trueness value of a model is also a prerequisite for reducing the time of operation, the duration of bleeding, and the postoperative morbidities of the patient [20]. In contrast, a slight room for compromise exists if a 3D model is printed for educational purposes where trueness is not a crucial requirement as that for surgical simulation or clinical scenario replication [14]. According to the findings of the current study, SLA and MJ offer a medium- and high-cost solution with a comparable mean absolute error of less than 0.1 mm, respectively, which could be considered as clinically acceptable for tasks involving treatment planning in craniomaxillofacial surgery. The low-cost FFF model showed an overall discrepancy of greater than 1 mm that might affect how pre-bent plates, surgical guides and implants fit. Furthermore, the longer printing time, up to 5 times longer than the medium- and high-cost printer, could further influence its efficiency in a 3D workflow, thereby, confirming its inapplicability for clinical applications. Nonetheless, it provides a practical and cost-effective solution for simulating procedures and anatomical education, as the printer was able to replicate the skeletal anatomy. It is also noteworthy that complexity of anatomical structures being printed should also be taken into consideration, as the craniomaxillary complex showed more deviation than mandible.

The low-cost FFF printer utilized in the present study showed a higher amount of discrepancy compared to other studies, whereas both consumer-grade and professional printers showed comparable trueness for printing skeletal models [7, 8, 16, 25]. The trueness of an FFF based model has been known to be mostly affected by the layer thickness and nozzle diameter. In this study, a nozzle size of $0.4 \text{mm}$ was used with a layer thickness of $0.15 \text{mm}$. The printing strategy, infill density and print orientation, along with the manufacturing parameters (extrusion temperature and bed temperature) might also play a role towards the model’s trueness [26]. The infill density applied in the present study was $15{\%}$, which was similar to the range of $10{\%}$ to $50{\%}$ reported in the previous studies [7, 12, 15, 16]. The manufacturing settings of $0.15 \text{mm}$ layer resolution, $200^\circ \text{C}$ extruder temperature and bed heat of $60^\circ \text{C}$, were in accordance with the settings proposed by Rendón-Medina et al. [17]. Even with the optimized settings, an increased error was observed specifically at the temporal region and skull base of the right and left skull respectively, which could have been due to the build orientation. Another source of error could have been induced by the removal of support structures at the post-processing step. In this context, it is also relevant to know that the use of high-end/professional FFF industrial 3D printers, featuring high throughput nozzle, temperature controlled closed chamber and/or higher axes resolution, could have facilitated the productions of more true models, albeit at higher cost with a longer print time [20]. Moreover, the impact of build orientation on the trueness of the models was not investigated, which should be considered in future studies.

The trueness of the SLA model was in accordance with other studies, where SLA was found to be optimal for fabricating skeletal structures with intricate details, given its superior resolution, ultimately related to the laser positional accuracy [27]. Likewise, MJ was able to optimally print the complex anatomical structures with a fine layer resolution and provided a better surface quality, which was also consistent with previous studies [27].

The main strength of the study was the first-time fabrication and trueness assessment of the craniomaxillary complex using a low-cost printer which has not been previously investigated. The findings highlight the potential of a low-cost printer for fabricating anatomical models which could be useful for treatment planning, surgical simulation, and anatomical education. However, it is crucial to conduct further testing and optimization before establishing their application in a routine clinical setting. The study also had certain limitations. Firstly, the study was limited to a small sample of three printers with different technologies and materials which cannot be generalized to all the printers. Secondly, the selection of material could have influenced the trueness. Hence, it is important to investigate the impact of different materials and additive components on material conversion and properties [26]. Thirdly, since just one normal skull was examined in this work, further research is required to determine how it relates to the pathological skull. Fourthly, owing to the small sample size and only trueness being evaluated, the findings of the study and statistical inference should be interpreted with caution. Future studies are recommended to assess the model’s accuracy with a larger sample size. Fifthly, the cost-effectiveness in this study was only based on the printer and the model price, thereby, further studies are also recommended to perform a detailed cost-analysis to include the costs related to electricity, maintenance, labor and license acquisition/renewal. Lastly, haptic feedback of the models for simulating surgical procedures was not assessed. In this respect, although the FFF and SLA material pallet is continuously enlarging, MJ technology is still the most viable option offering the largest range of materials for simulating soft and hard tissue.

## Conclusions

Both stereolithography and multi-jetting were able to replicate the skeletal anatomy on a medium- and high-cost printer, respectively, with the least amount of error, thereby confirming their applicability for clinical application, such as pre-bending plates and fabricating implants. Desktop/consumer grade FFF printer offered the highest discrepancy which might not be optimal for clinical applications, however, it could serve as a cost-effective alternative for surgical simulation, anatomical education, and/or patient communication.

## Data Availability

The datasets generated and/or analysed during the current study are available in the KU Leuven Internet File repository, the data that support the findings of this study are available from the corresponding author upon reasonable request.

## Abbreviations

3D: Three-dimensional
ABS: Acrylonitrile butadiene styrene
AM: Additive manufacturing
ANOVA: Analysis of variance
CBCT: Cone-beam computed tomography
DICOM: Digital Imaging and Communications in Medicine
FFF: Fused Filament Fabrication
MAE: Mean absolute error
MJ: Material Jetting
PLA: Polylactic acid
RMS: Root mean square
RVD: Relative volume difference
RP: Rapid prototyping
SLA: Stereolithography
SLM: Selective laser melting
SLS: Selective laser sintering
STL: Standard tessellation language
UV: Ultraviolet
